# METTL14-Mediated lncRNA MSTRG.292666.16 m^6^A Modification Promotes the Progression of Non-Small-Cell Lung Cancer Through the MAPK Pathway

**DOI:** 10.3390/ijms27177868

**Published:** 2026-09-03

**Authors:** Qinfang Deng, Hui Sun, Heyong Wang, Chenlei Cai, Xianxiu Ji, Qiyu Fang, Boxiong Xie, Songwen Zhou

**Affiliations:** 1 Department of Oncology, Shanghai Pulmonary Hospital, School of Medicine, Tongji University, Shanghai 200433, China; 2 Central Laboratory, Shanghai Pulmonary Hospital, School of Medicine, Tongji University, Shanghai 200433, China; 3 Department of Thoracic, Shanghai Pulmonary Hospital, School of Medicine, Tongji University, Shanghai 200433, China

**Keywords:** non-small-cell lung cancer, m^6^A modification, METTL14, lncRNA MSTRG.292666.16, MAPK pathway

## Abstract

Non-small-cell lung cancer (NSCLC) treatment is hampered by its complex pathogenesis and high heterogeneity. N6-methyladenosine (m^6^A) represents the most common post-transcriptional modification regulating RNA stability and function in eukaryotes. This methylation is catalyzed by methyltransferase complexes, with METTL14 being the core catalytic subunit. Abnormal expression of lncRNA *MSTRG.292666.16* is related to poor prognosis of NSCLC. However, the mechanism by which it regulates NSCLC progression through m^6^A modification remains unclear. We employed cell function experiments, molecular mechanism analysis, RNA interaction experiments, and a nude mouse tumor model to explore the roles of METTL14-mediated *MSTRG.292666.16* m^6^A modification in NSCLC and the potential MAPK signaling pathway involved. *METTL14* was significantly upregulated in NSCLC cell lines and promoted m^6^A modification of *MSTRG.292666.16* by forming a stable association with it. *METTL14* knockdown significantly inhibited the viability, migration and invasion of A549 cells and promoted apoptosis, whereas *MSTRG.292666.16* overexpression reversed these effects. Mechanistically, METTL14 upregulated the expression of *MSTRG.292666.16* through m^6^A modification, thereby activating the MAPK pathway (manifested as elevated levels of MAPK8IP3 and p-ERK1/2). The use of a selective p38 MAPK inhibitor SB203580 stimulated the tumor-suppressive effect of *METTL14* knockdown, whereas the activator U-46619 reversed it. In vivo experiments confirmed that *METTL14* knockdown significantly inhibited tumor growth, whereas *MSTRG.292666.16* overexpression partially restored the malignant phenotype of the tumor, which was associated with MAPK pathway activation. This study revealed that *METTL14*-dependent m^6^A modification of *MSTRG.292666.16* may act as an upstream driver to activate the MAPK cascade and facilitate NSCLC progression. These findings clarify a key epitranscriptomic regulatory mechanism driving NSCLC development and offer preliminary molecular clues for exploring potential therapeutic targets in subsequent clinical NSCLC research.

## 1. Introduction

Non-small-cell lung cancer (NSCLC) is one of the leading causes of cancer-related deaths worldwide that poses significant clinical challenges owing to its complex pathogenesis and high heterogeneity [1]. Epidemiologically, NSCLC currently accounts for approximately 85% of all lung cancer cases, with five-year survival rate of less than 20% [2,3]. Surgery, chemotherapy, and radiotherapy are the conventional treatment approaches; however, for patients with advanced disease, surgery alone is insufficient in suppressing cancer progression [4]. With continuous advancements in genetic testing technologies, epidermal growth factor receptor tyrosine kinase inhibitors (EGFR-TKIs) offer a critical modality for precision therapy in NSCLC [5]. Although targeted therapies and immunotherapies have significantly improved the outcomes for some patients in recent years, the emergence of drug resistance as well as tumor metastasis and recurrence still lead to suboptimal overall survival rates for patients with NSCLC, particularly those in advanced stage of the disease [6,7]. Therefore, more in-depth exploration of the molecular mechanisms underlying NSCLC development and progression, along with the identification of novel therapeutic targets and biomarkers, holds crucial clinical significance.

Long non-coding RNAs (lncRNAs) are a class of non-coding RNA molecules exceeding 200 nucleotides in length, which have been demonstrated in recent years to participate in tumorigenesis and cancer progression through diverse mechanisms, including gene expression regulation, epigenetic modification, and signaling pathway activation [8,9]. Chen et al. [10] reported significant downregulation of lncRNA *SLCO4A1-AS1* in lung cancer cells and in patients with NSCLC. They also showed that *SLCO4A1-AS1* could inhibit lung cancer development by antagonizing TOX4/NTSR1 signaling pathway. We previously reported that lncRNA *MSTRG.292666.16* levels in plasma-derived exosomes were significantly higher in osimertinib-resistant patients than in their sensitive counterparts and that exosomal lncRNA *MSTRG.292666.16* could promote acquired resistance to osimertinib, thereby playing a pivotal role in mediating resistance transmission [11]. Additionally, M2-polarized tumor-associated macrophage-derived exosomes were reported to drive osimertinib resistance in NSCLC via the lncRNA *MSTRG.292666.16*/*miR-6386-5p*/MAPK8IP3 regulatory axis [12]. These reports indicate that lncRNA *MSTRG.292666.16* may be involved in drug resistance and may serve as a therapeutic target for NSCLC. However, the precise molecular mechanisms underlying the role of lncRNA *MSTRG.292666.16* in NSCLC remain largely elusive, particularly with regard to how its post-transcriptional modifications regulate biological functions, warranting further investigation.

N6-methyladenosine (m^6^A) modification is one of the most prevalent epitranscriptomic modifications in eukaryotic RNA, which is catalyzed by methyltransferase complexes (such as METTL3/METTL14/WTAP) [13]. Beyond its role in mRNA regulation, m^6^A modification governs the biogenesis and function of lncRNAs [14]. The impact of m^6^A on lncRNAs may involve multiple regulatory mechanisms. On one hand, m^6^A modification may modulate lncRNA function by providing binding sites for m^6^A reader proteins or by altering local RNA structure to facilitate the recruitment of RNA-binding proteins. On the other hand, it may also influence lncRNA–DNA interactions by affecting RNA–DNA triplex formation [14,15,16]. As core components of the m^6^A methyltransferase complex, METTL3, METTL14, and WTAP play critical roles in regulating the initiation and progression of various cancers [17]. For example, Sun et al. [18] found that METTL3 was not only a prognostic marker associated with unfavorable outcomes in small-cell lung cancer (SCLC), but also drove resistance to chemotherapy by inducing mitochondrial autophagy. The loss of METTL14 is associated with a poor prognosis in patients with colorectal cancer (CRC), and this protein can suppress the CRC proliferation and metastasis by downregulating lncRNA *XIST* [19]. Furthermore, WTAP was reported to be highly expressed in hepatocellular carcinoma (HCC) and to promote HCC progression through m^6^A-HuR-dependent epigenetic silencing of ETS1 [20]. However, whether METTL14/METTL3/WTAP regulate the progression of NSCLC by mediating the m^6^A modification of lncRNA *MSTRG.292666.16* remains unclear.

The mitogen-activated protein kinase (MAPK) cascade governs core cellular events, including proliferation, differentiation and apoptosis, and its persistent hyperactivation fuels NSCLC cell proliferation, anti-apoptosis and chemoresistance [21,22,23]. In preliminary and published studies, we found that lncRNA *MSTRG.292666.16* drives osimertinib resistance in NSCLC specifically through the *MSTRG.292666.16*/*miR-6836-5p*/MAPK8IP3 regulatory axis [12], and that this lncRNA is highly expressed in NSCLC and is linked to an unfavorable prognosis [24]. Moreover, our group previously published preliminary evidence, based solely on A549 cell line and simple xenograft tumor growth curves, confirming that METTL14-mediated m^6^A modification upregulates lncRNA *MSTRG.292666.16* to drive NSCLC malignant phenotypes [24]. Nevertheless, this preliminary study did not provide direct evidence for METTL14–*MSTRG.292666.16* binding and lacked downstream signaling pathway exploration, independent cellular validation, and systematic detection of m^6^A regulators and tumor tissue biomarkers. We sought to overcome these limitations in this study. We cross-validated the previous findings in A549 and NCI-H1299 cells, performed RNA pull-down, RNA immunoprecipitation (RIP) and MeRIP-qPCR to verify the association between METTL14 and *MSTRG.292666.16*, identified MAPK8IP3/MAPK cascade as the core downstream effector via pharmacological rescue assays, characterized the changes in the expression of m^6^A readers/erasers, and supplemented the findings with multiple pathological and molecular experiments using xenograft tissues. Furthermore, we anticipated potential reciprocal feedback between activated MAPK and cellular m^6^A regulatory machinery to form a self-sustaining oncogenic regulatory circuit, which we sought to characterize via pharmacological pathway modulation. This in vitro and in vivo mechanistic study was aimed at elucidating the regulatory network connecting METTL14, lncRNA *MSTRG.292666.16*, and the MAPK pathway and offers preliminary theoretical molecular insights that may lay the foundation for subsequent clinical translational research on therapeutic targets for NSCLC.

## 2. Results

### 2.1. Expression Analysis of m^6^A-Related Genes in NSCLC Cells, and Their Interaction with lncRNA MSTRG.292666.16

**Figure 1:**
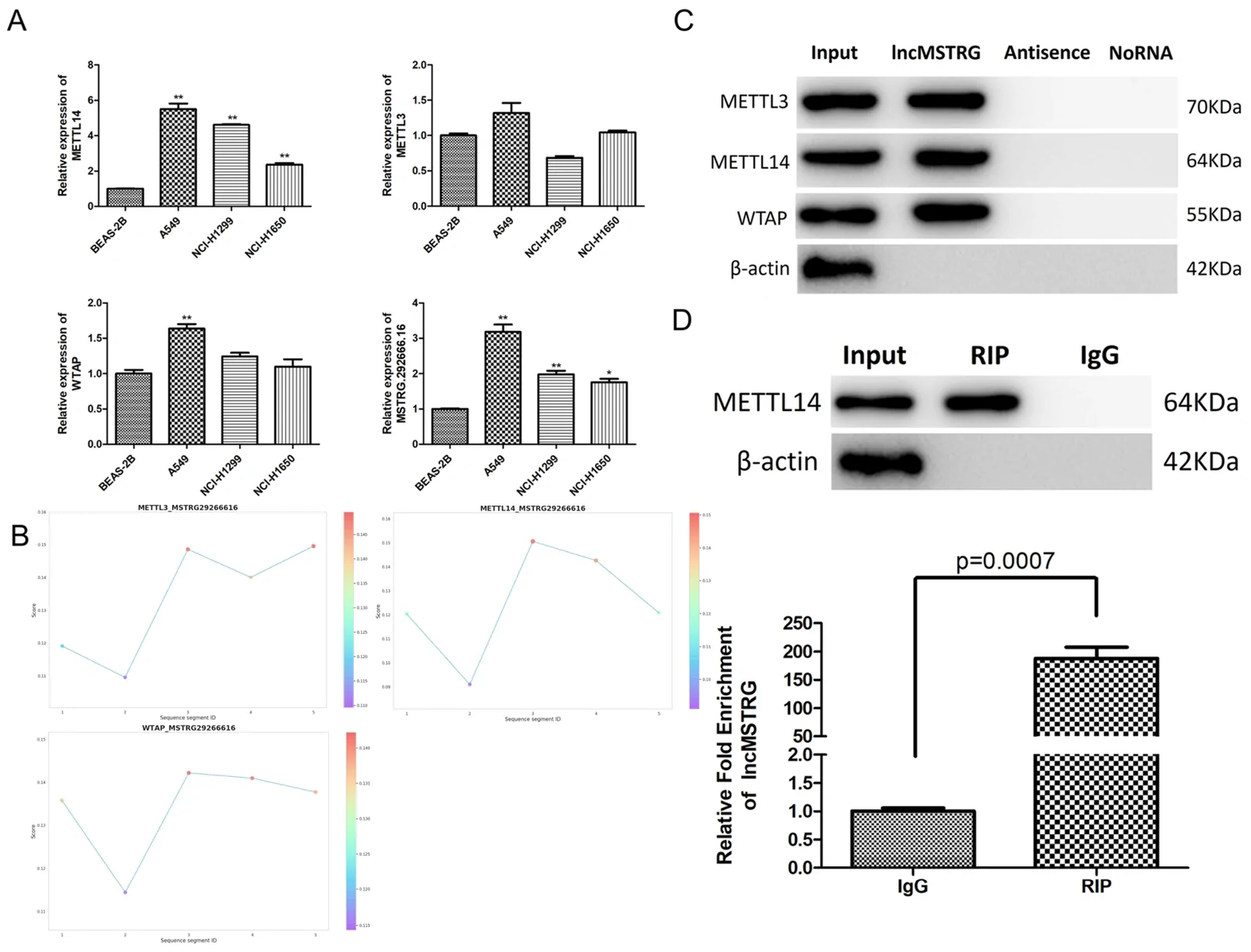
Evaluation of the expression of m^6^A-related genes in NSCLC cells and their interaction with lncRNA *MSTRG.292666.16*. (**A**) The relative expression of *METTL14*, *METTL3*, *WTAP*, and *MSTRG.292666.16* in different NSCLC cell lines (A549, NCI-H1299, and NCI-H1650) measured using RT-qPCR. (**B**) RBPsuite analysis software predicted *MSTRG.292666.16* to bind to m^6^A-related genes (*METTL3*, *METTL14*, and *WTAP*). (**C**) RNA pull-down verified the specific associations between *MSTRG.292666.16* and m^6^A-related proteins (METTL3, METTL14, and WTAP). (**D**) RIP experiments were conducted using the METTL14 antibody, and relative fold enrichment of lncRNA *MSTRG.292666.16* was determined to be 187.82, which confirmed a prominent association between lncRNA *MSTRG.292666.16* and METTL14 in A549 cells. * *p* < 0.05, ** *p* < 0.01, vs. the BEAS-2B cells.

To explore the m^6^A modification in NSCLC, the expression levels of m^6^A-related genes were estimated in NSCLC cell lines (A549, NCI-H1299, and NCI-H1650). *METTL14* expression was significantly upregulated in all the NSCLC cell lines (A549, NCI-H1299, and NCI-H1650) compared with that in BEAS-2B cells (*p* < 0.01, Figure 1A). However, no significant differences in *METTL3* expression were noted among the BEAS-2B, A549, NCI-H1299, and NCI-H1650 cells (*p* > 0.05, Figure 1A). Among the NSCLC cell lines, the expression of *WTAP* was evidently higher only in A549 cells than in BEAS-2B cells (*p* < 0.01, Figure 1A). Using the RBPsuite analysis software (http://www.csbio.sjtu.edu.cn/bioinf/RBPsuite/; Access date: 31 December 2024), lncRNA *MSTRG.292666.16* was predicted to bind to m^6^A-related genes (*METTL3*, *METTL14*, and *WTAP*) (Figure 1B). In RNA pull-down followed by Western blotting of the precipitated proteins, lncRNA *MSTRG.292666.16* was found to form specific associations with m^6^A-related proteins (METTL3, METTL14, and WTAP) (Figure 1C). Additionally, considering the high expression of METTL14 in all NSCLC cell lines, we further investigated the association between METTL14 and lncRNA *MSTRG.292666.16* via RIP with the METTL14 antibody. The relative fold enrichment of lncRNA *MSTRG.292666.16* in A549 cells was 187.82 after METTL14 RIP (Figure 1D), which further verified the robust association between METTL14 and lncRNA *MSTRG.292666.16*. Based on these results, we selected METTL14 and A549 cells for subsequent experiments.

### 2.2. Roles of METTL14 and lncRNA MSTRG.292666.16 in the Growth of A549 Cells

**Figure 2:**
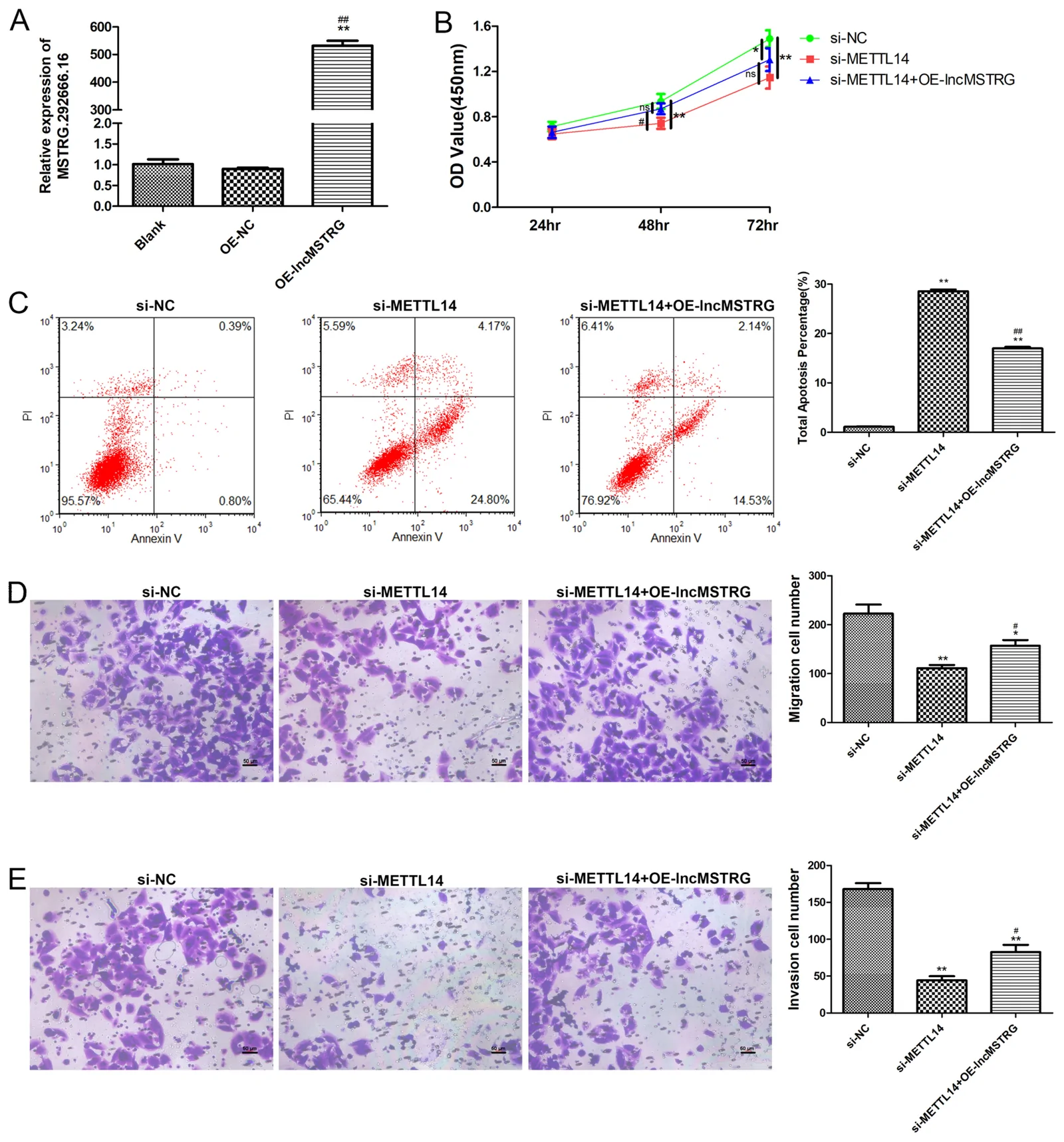
Roles of *METTL14*/lncRNA *MSTRG.292666.16* in the growth of A549 cells and related molecular mechanisms. (**A**) Cell transfection efficiency evaluated by determining the expression of *MSTRG.292666.16* using RT-qPCR. ** *p* < 0.01, vs. blank; ## *p* < 0.01, vs. OE-NC. (**B**) Viability of A549 cells after *METTL14* knockdown and *MSTRG.292666.16* overexpression, assessed using the CCK-8 assay. (**C**) Representative flow cytometry dot plots and histograms for quantitative analyses of apoptosis in A549 cells after *METTL14* knockdown and *MSTRG.292666.16* overexpression. The cell migration (**D**) and invasion (**E**) of A549 cells after *METTL14* knockdown and *MSTRG.292666.16* overexpression, assessed using Transwell assay. * *p* < 0.05, ** *p* < 0.01, vs. si-NC; # *p* < 0.05, ## *p* < 0.01, vs. si-*METTL14*.

To investigate the roles of *METTL14*/lncRNA *MSTRG.292666.16* in NSCLC cells, A549 cells overexpressing *MSTRG.292666.16* were constructed. No significant difference was observed in *MSTRG.292666.16* expression between the blank and OE-NC groups (*p* > 0.05); OE-lncRNA transfection evidently increased the expression of *MSTRG.292666.16* compared with that in the blank A549 cells (*p* < 0.01, Figure 2A). These results confirmed the successful establishment of *MSTRG.292666.16*-overexpressing A549 cells.

We evaluated the viability, apoptosis, migration, and invasion of A549 cells subjected to different treatments. The viability of cells in different treatment groups gradually increased with the time in culture (Figure 2B). When cultured for 48 and 72 h, *METTL14* knockdown significantly inhibited cell viability relative to that in the si-NC group (*p* < 0.01), whereas *MSTRG.292666.16* overexpression markedly enhanced the viability of A549 cells caused by *METTL14* knockdown (*p* < 0.05, Figure 2B). In comparison with the si-NC–transfected A549 cells, *METTL14* silencing enhanced cell apoptosis (*p* < 0.01), whereas *MSTRG.292666.16* overexpression reversed the effects of *METTL14* silencing on the apoptosis of A549 cells (*p* < 0.01, Figure 2C). Cell migration and invasion were significantly decreased in A549 cells transfected with si-*METTL14* compared with that in the si-NC cells (*p* < 0.01), and the decrease was reversed by *MSTRG.292666.16* overexpression compared with that for the si-*METTL14*-treated cells (*p* < 0.05, Figure 2D,E). These results indicated that *METTL14* knockdown significantly inhibited the viability, migration, and invasion of A549 cells, while inducing apoptosis.

### 2.3. Roles of METTL14/lncRNA MSTRG.292666.16 in the Expression of MAPK-Related Proteins and m^6^A-Related Proteins

**Figure 3:**
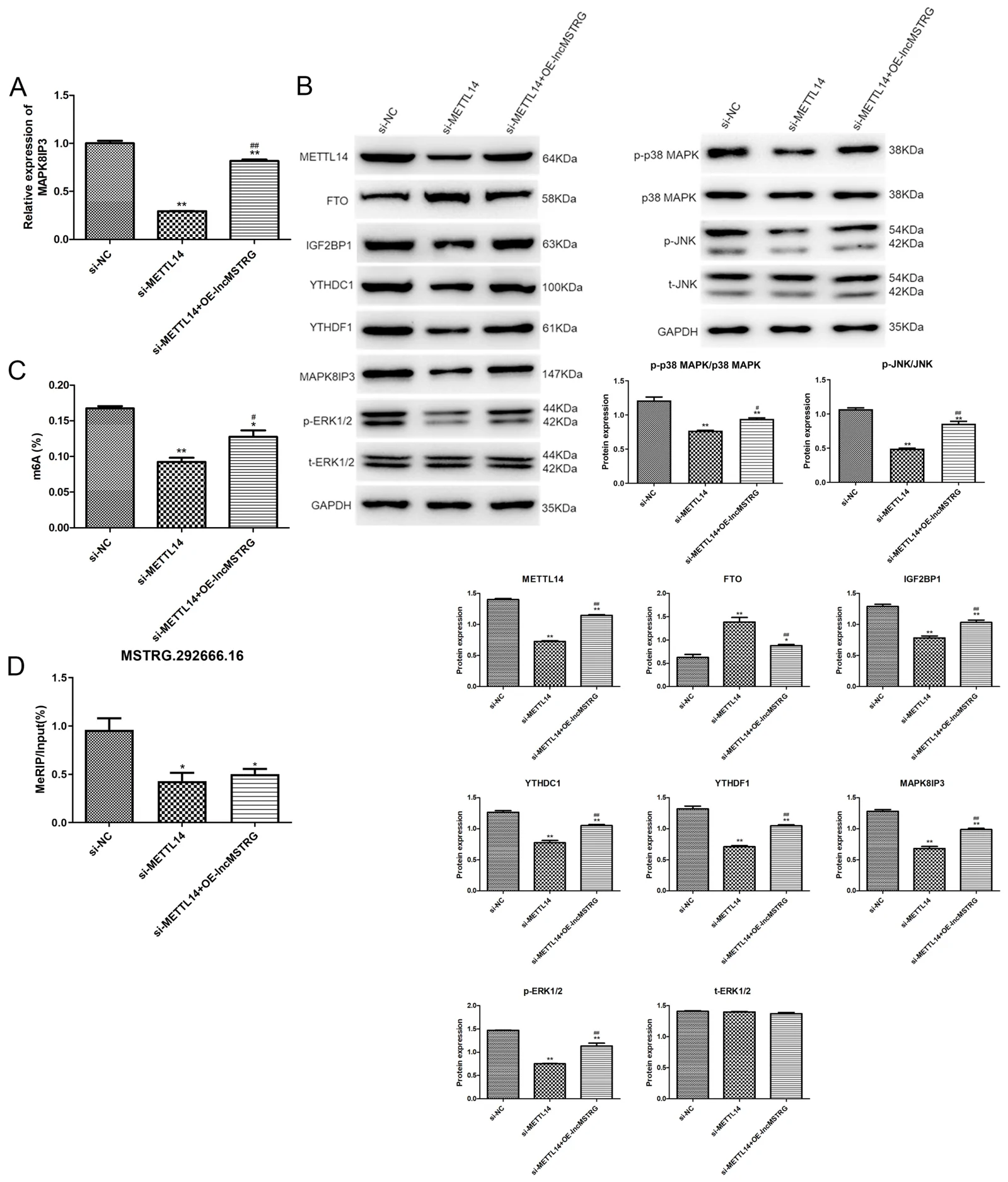
Effects of *METTL14* and lncRNA *MSTRG.292666.16* on the expression of related molecules in A549 cells. (**A**) The relative expression of *MAPK8IP3* in A549 cells after *METTL14* knockdown and *MSTRG.292666.16* overexpression, assessed using RT-qPCR. (**B**) The protein expression of MAPK-related markers (MAPK8IP3, p-ERK1/2, t-ERK1/2, p-p38 MAPK, p38 MAPK, p-JNK, and t-JNK) and m^6^A-related proteins (METTL14, FTO, IGF2BP1, YTHDC1, and YTHDF1) in A549 cells after *METTL14* knockdown and *MSTRG.292666.16* overexpression, assessed using Western blotting. (**C**) The global m^6^A levels in A549 cells after *METTL14* knockdown and *MSTRG.292666.16* overexpression, evaluated using an m^6^A RNA Methylation Quantification Kit. (**D**) The m^6^A levels in A549 cells after *METTL14* knockdown and *MSTRG.292666.16* overexpression, evaluated using MeRIP-qPCR. * *p* < 0.05, ** *p* < 0.01, vs. si-NC; # *p* < 0.05, ## *p* < 0.01, vs. si-*METTL14*.

To determine the molecular mechanisms underlying the regulation of NSCLC cell growth by *METTL14*/lncRNA *MSTRG.292666.16*, the expression of MAPK-related markers (MAPK8IP3, p-ERK1/2, t-ERK1/2, p-p38 MAPK, p38 MAPK, p-JNK, and t-JNK) and m^6^A-related proteins (METTL14, FTO, IGF2BP1, YTHDC1, and YTHDF1) was detected using RT-qPCR and Western blotting. RT-qPCR showed the *MAPK8IP3* expression was significantly lower after *METTL14* silencing than that in si-NC cells (*p* < 0.01), whereas it was evidently higher in the si-*METTL14*+OE-lncMSTRG group than that in the si-*METTL14* group (*p* < 0.01, Figure 3A). Western blotting analyses revealed altered abundance of m^6^A regulatory proteins following *METTL14* depletion: IGF2BP1, YTHDC1, and YTHDF1 levels were markedly decreased, whereas those of the m^6^A eraser FTO were elevated (*p* < 0.01). Ectopic *MSTRG.292666.16* overexpression partially restored the levels of these proteins in *METTL14*-knockdown cells (*p* < 0.01, Figure 3B). The trend of MAPK8IP3 protein expression in different groups, as detected using Western blotting, was consistent with that of its mRNA determined using RT-qPCR (Figure 3B). No significant differences in t-ERK1/2 protein expression were noted among the si-NC, si-*METTL14*, and si-*METTL14*+OE-lncMSTRG groups (*p* > 0.05). However, p-ERK1/2 levels after *METTL14* knockdown were significantly reduced compared with those in the si-NC-transfected A549 cells (*p* < 0.01), and this was reversed by *MSTRG.292666.16* overexpression (*p* < 0.01, Figure 3B). Additionally, the protein levels of p-p38 MAPK/p38 MAPK and p-JNK/JNK were significantly reduced in A549 cells with *METTL14* knockdown compared with those in the si-NC group (*p* < 0.01); the *MSTRG.292666.16* overexpression in A549 cells enhanced the protein levels (*p* < 0.05, Figure 3B).

Finally, m^6^A levels in cells subjected to different treatments were measured. The global m^6^A levels in A549 cells with *METTL14* knockdown were significantly reduced compared with those in the control cells (*p* < 0.01); however, *MSTRG.292666.16* overexpression evidently restored the m^6^A levels decreased by *METTL14* knockdown (*p* < 0.05, Figure 3C). To directly quantify the abundance of m^6^A modification on *MSTRG.292666.16* transcripts, we performed MeRIP-qPCR. *METTL14* silencing markedly reduced the fold enrichment of m^6^A on *MSTRG.292666.16* (*p* < 0.05). Notably, ectopic overexpression of lncRNA *MSTRG.292666.16* failed to restore the diminished m^6^A modification of *MSTRG.292666.16* induced by *METTL14* knockdown in the si-*METTL14*+OE-lncMSTRG group (*p* > 0.05, Figure 3D), which validated that the notion of m^6^A modification of *MSTRG.292666.16* is *METTL14*-dependent.

### 2.4. In Vitro Validation of the Role of METTL14/lncRNA MSTRG.292666.16 in Another NSCLC Cell Line (NCI-H1299)

**Figure 4:**
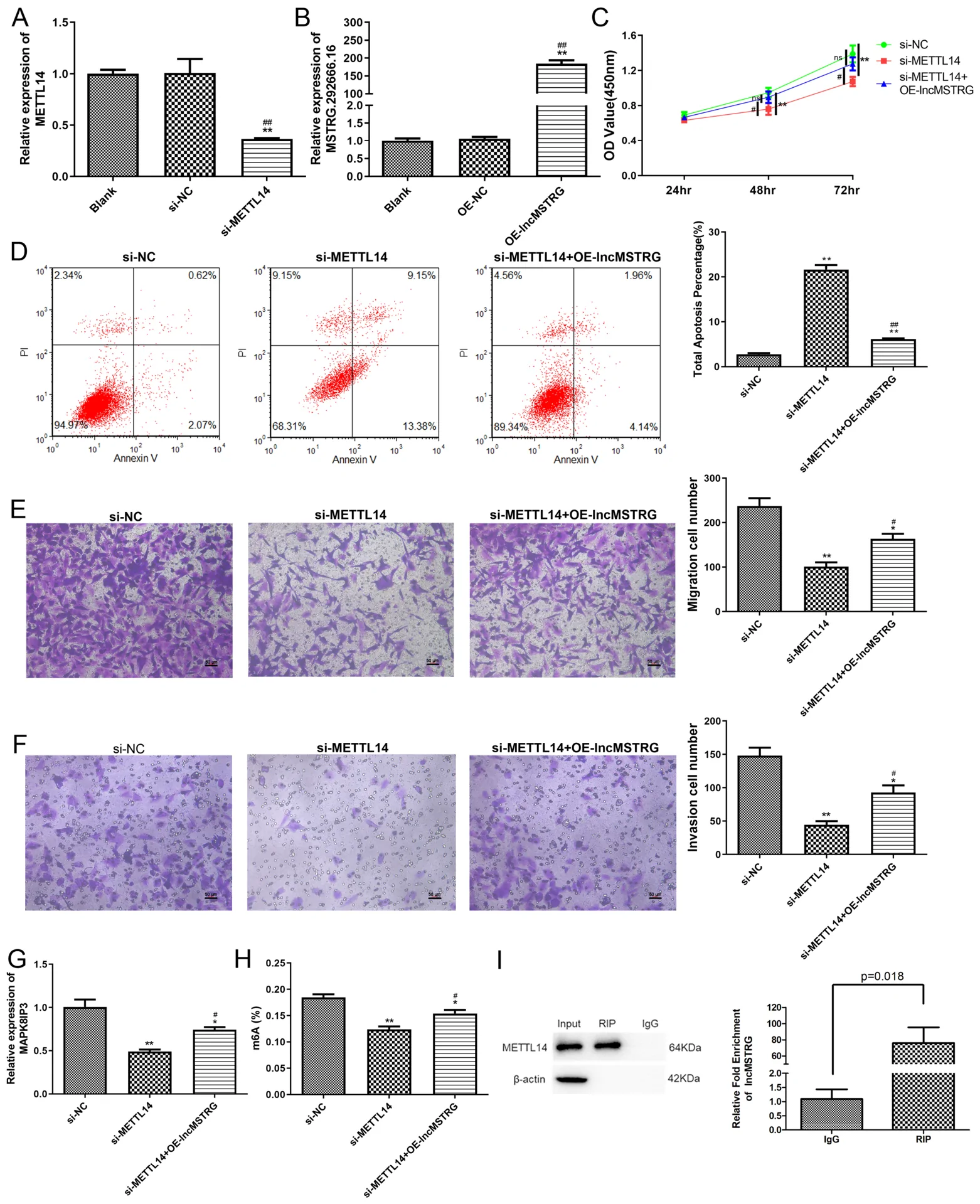
Validation of the roles of *METTL14* and lncRNA *MSTRG.292666.16* in growth of another NSCLC cell line NCI-H1299. (**A**) Cell transfection efficiency assessed by measuring the expression of *METTL14* using RT-qPCR. ** *p* < 0.01, vs. blank; ## *p* < 0.01, vs. si-NC. (**B**) Cell transfection efficiency evaluated by determining the expression of *MSTRG.292666.16* using RT-qPCR. ** *p* < 0.01, vs. blank; ## *p* < 0.01, vs. OE-NC. (**C**) Viability of NCI-H1299 cells after *METTL14* knockdown and *MSTRG.292666.16* overexpression, assessed using the CCK-8 assay. (**D**) Representative flow cytometry dot plots and histograms for quantitative analyses of apoptosis in NCI-H1299 cells after *METTL14* knockdown and *MSTRG.292666.16* overexpression. The cell migration (**E**) and invasion (**F**) of NCI-H1299 cells after *METTL14* knockdown and *MSTRG.292666.16* overexpression, assessed using Transwell assay. (**G**) The relative expression of *MAPK8IP3* in NCI-H1299 cells after *METTL14* knockdown and *MSTRG.292666.16* overexpression, assessed using RT-qPCR. (**H**) The global m^6^A levels in NCI-H1299 cells after *METTL14* knockdown and *MSTRG.292666.16* overexpression, assessed using an m^6^A RNA Methylation Quantification Kit. (**I**) Confirmation of the association between lncRNA *MSTRG.292666.16* and *METTL14* in NCI-H1299 cells using RIP experiments. * *p* < 0.05, ** *p* < 0.01, vs. si-NC; # *p* < 0.05, ## *p* < 0.01, vs. si-*METTL14*.

To further validate the role of *METTL14*/lncRNA *MSTRG.292666.16* in NSCLC, we used another NSCLC cell line NCI-H1299. In NCI-H1299 cells, *METTL14* knockdown significantly downregulated *METTL14* expression (*p* < 0.01, Figure 4A), and lncRNA *MSTRG.292666.16* upregulated the *MSTRG.292666.16* expression (*p* < 0.01, Figure 4B). These results indicated the successful generation of NCI-H1299 cells with *METTL14* silencing or *MSTRG.292666.16* overexpression.

CCK8 results indicated that prolonged cell culture led to a time-dependent increase in cell viability for every treatment cohort (Figure 4C). Relative to the si-NC group, *METTL14* knockdown drastically reduced the viability of A549 cells at 48 and 72 h of culture (*p* < 0.01). Conversely, *MSTRG.292666.16* overexpression substantially restored the proliferative capacity that was suppressed by *METTL14* knockdown (*p* < 0.05, Figure 4C). Compared with the si-NC NCI-H1299 cells, *METTL14* knockdown remarkably enhanced apoptosis (*p* < 0.01), whereas *MSTRG.292666.16* overexpression fully counteracted the proapoptotic effects triggered by *METTL14* silencing (*p* < 0.01, Figure 4D). With regard to the migration and invasion of NCI-H1299 cells, fewer migrated and invaded cells were observed in si-*METTL14*-transfected NCI-H1299 cells relative to those in the si-NC group (*p* < 0.01). Notably, ectopic overexpression of *MSTRG.292666.16* substantially restored the number of migratory and invasive cells suppressed by *METTL14* knockdown (*p* < 0.05, Figure 4E,F).

RT-qPCR quantification revealed that *MAPK8IP3* transcript levels were markedly downregulated upon *METTL14* knockdown relative to the levels in si-NC cells (*p* < 0.01), whereas NCI-H1299 cells cotransfected with si-*METTL14* and the *MSTRG.292666.16* overexpression plasmid exhibited drastically increased *MAPK8IP3* expression compared with that in the sole si-*METTL14* group (*p* < 0.05, Figure 4G). Global m^6^A modification levels were quantified in all treated cell groups. *METTL14* knockdown triggered a remarkable reduction in total m^6^A abundance in NCI-H1299 cells relative to that in si-NCs (*p* < 0.01). In contrast, ectopic overexpression of *MSTRG.292666.16* effectively rescued the m^6^A deficiency caused by *METTL14* silencing (*p* < 0.05, Figure 4H). As evident from RIP analysis of NCI-H1299 cells, lncRNA *MSTRG.292666.16* exhibited a relative enrichment fold of 76 upon METTL14 pulldown (Figure 4I), further supporting the association between METTL14 and lncRNA *MSTRG.292666.16* in NCI-H1299 cells.

### 2.5. Effects of METTL14/lncRNA MSTRG.292666.16 on Subcutaneous Xenograft Tumor Growth in Nude Mice

**Figure 5:**
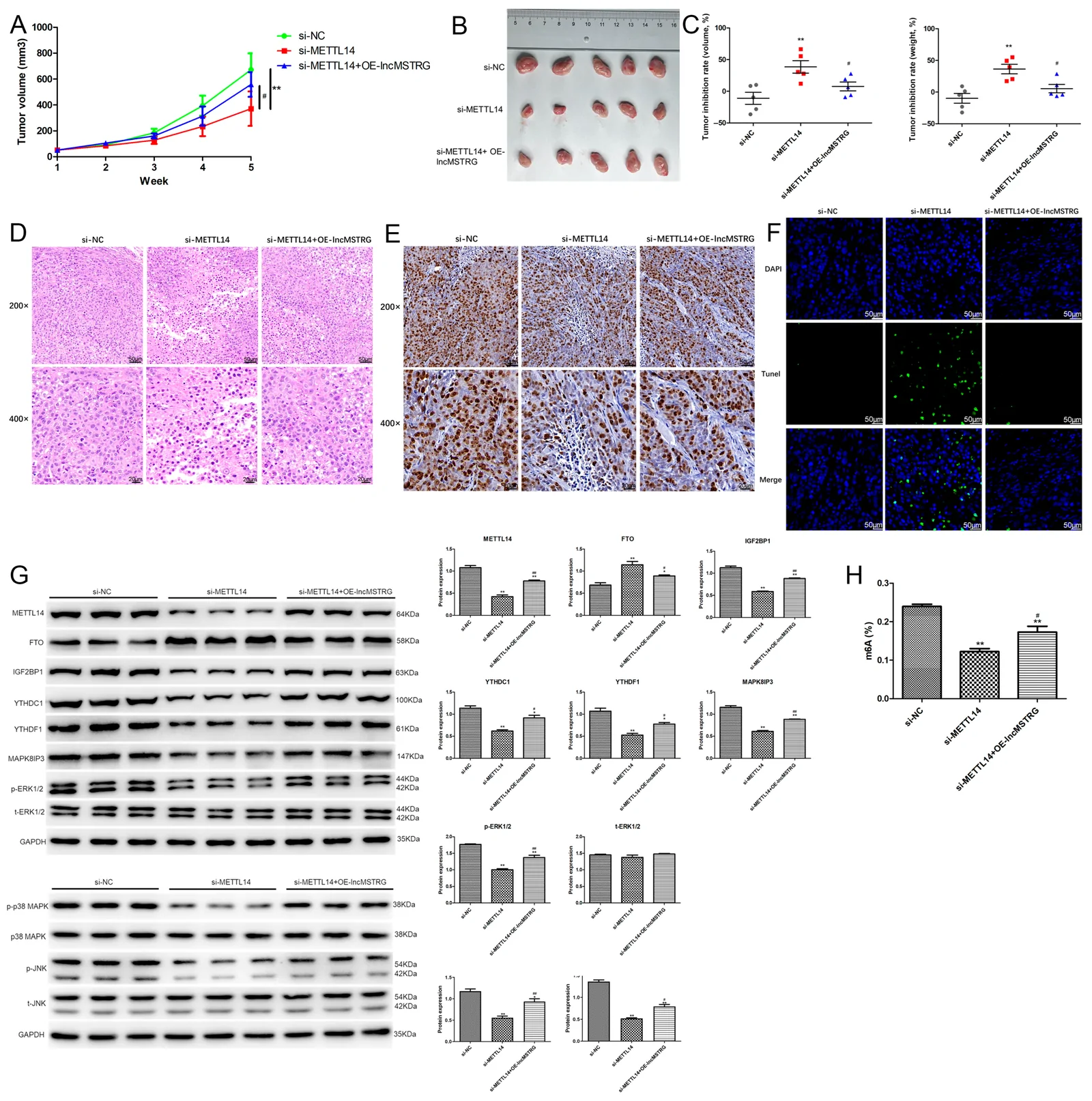
Effects of *METTL14* and lncRNA *MSTRG.292666.16* on subcutaneous xenograft tumor growth in nude mice. (**A**) The growth curve for the tumors in nude mice after tumor formation. (**B**) The size of tumor in different mice. (**C**) The tumor inhibition rate, assessed as percentage change in the volume and weight of tumors in different mice groups. (**D**) HE staining of the lung tissue. (**E**) Ki67 staining of the lung tissue. (**F**) Apoptosis of tumor tissues measured using TUNEL staining. (**G**) The protein expression of MAPK-related markers (MAPK8IP3, p-ERK1/2, t-ERK1/2, p-p38 MAPK, p38 MAPK, p-JNK, and t-JNK) and m^6^A-related proteins (METTL14, FTO, IGF2BP1, YTHDC1, and YTHDF1) in different mice groups, assessed using Western blotting. (**H**) The global m^6^A levels in different mice groups. * *p* < 0.05, ** *p* < 0.01, vs. si-NC; # *p* < 0.05, ## *p* < 0.01, vs. si-*METTL14*.

In vivo experiments showed that the tumor volume increased with increasing time, and at week 5, knockdown of *METTL14* significantly decreased the tumorigenesis ability of A549 cells in nude mice compared with that in the si-NC group (*p* < 0.01); *MSTRG.292666.16* overexpression promoted the tumorigenesis ability of A549 cells caused by *METTL14* knockdown (*p* < 0.05, Figure 5A). Compared with that in the si-NC group, the tumor size decreased significantly after *METTL14* knockdown, and the tumor inhibition rate exhibited an extremely significant increase (*p* < 0.01), whereas the tumor size increased significantly after *MSTRG.292666.16* overexpression, and the tumor inhibition rate significantly decreased compared with that in the si-*METTL14* group (*p* < 0.05, Figure 5B,C). HE staining showed clear morphology of cells in the si-NC group, with distinct boundaries and the cells neatly arranged. After *METTL14* knockdown, the intercellular structure was damaged and the cells were disordered. However, the morphology of cells in the si-*METTL14*+OE-lncMSTRG group was similar to that in the si-NC group (Figure 5D). Additionally, after *METTL14* knockdown, the proliferation of the tumor was significantly reduced, whereas the apoptosis was evidently enhanced relative to that in the si-NC group; overexpression of *MSTRG.292666.16* significantly promoted the proliferation of tumor and suppressed the apoptosis induced by *METTL14* knockdown (Figure 5E,F).

We next assessed the expression of m^6^A-related proteins (METTL14, FTO, IGF2BP1, YTHDC1, and YTHDF1) and MAPK-related proteins (MAPK8IP3, p-ERK1/2, t-ERK1/2, p-p38 MAPK, p38 MAPK, p-JNK, and t-JNK) in different tumor tissues via Western blotting. Compared with the si-NC group, *METTL14* knockdown significantly downregulated METTL14, IGF2BP1, YTHDC1, and YTHDF1 but upregulated FTO expression (*p* < 0.01); however, MSTRG.292666.16 overexpression markedly reversed the changes in expression induced by METTL14 knockdown (*p* < 0.05, Figure 5G). For MAPK-related proteins, no significant changes were observed in the expression of the t-ERK1/2 protein among the si-NC, si-*METTL14*, and si-*METTL14*+OE-lncMSTRG groups (*p* > 0.05). However, the expression trend of MAPK8IP3 and p-ERK1/2 protein in the different groups was similar to that of METTL14 (Figure 5G). Moreover, the trend of p-p38 MAPK/p38 MAPK and p-JNK/JNK expression in the different groups was similar to that of the p-ERK1/2 protein expression (Figure 5G). Ultimately, after *METTL14* knockdown, the global m^6^A levels in the lung tissue were significantly decreased compared with those in mice administered the si-NC-treated A549 cells (*p* < 0.01), whereas they were significantly increased upon *MSTRG.292666.16* overexpression (*p* < 0.05, Figure 5H).

### 2.6. METTL14 Regulated NSCLC Cell Growth Through the MAPK Signaling Pathway

**Figure 6:**
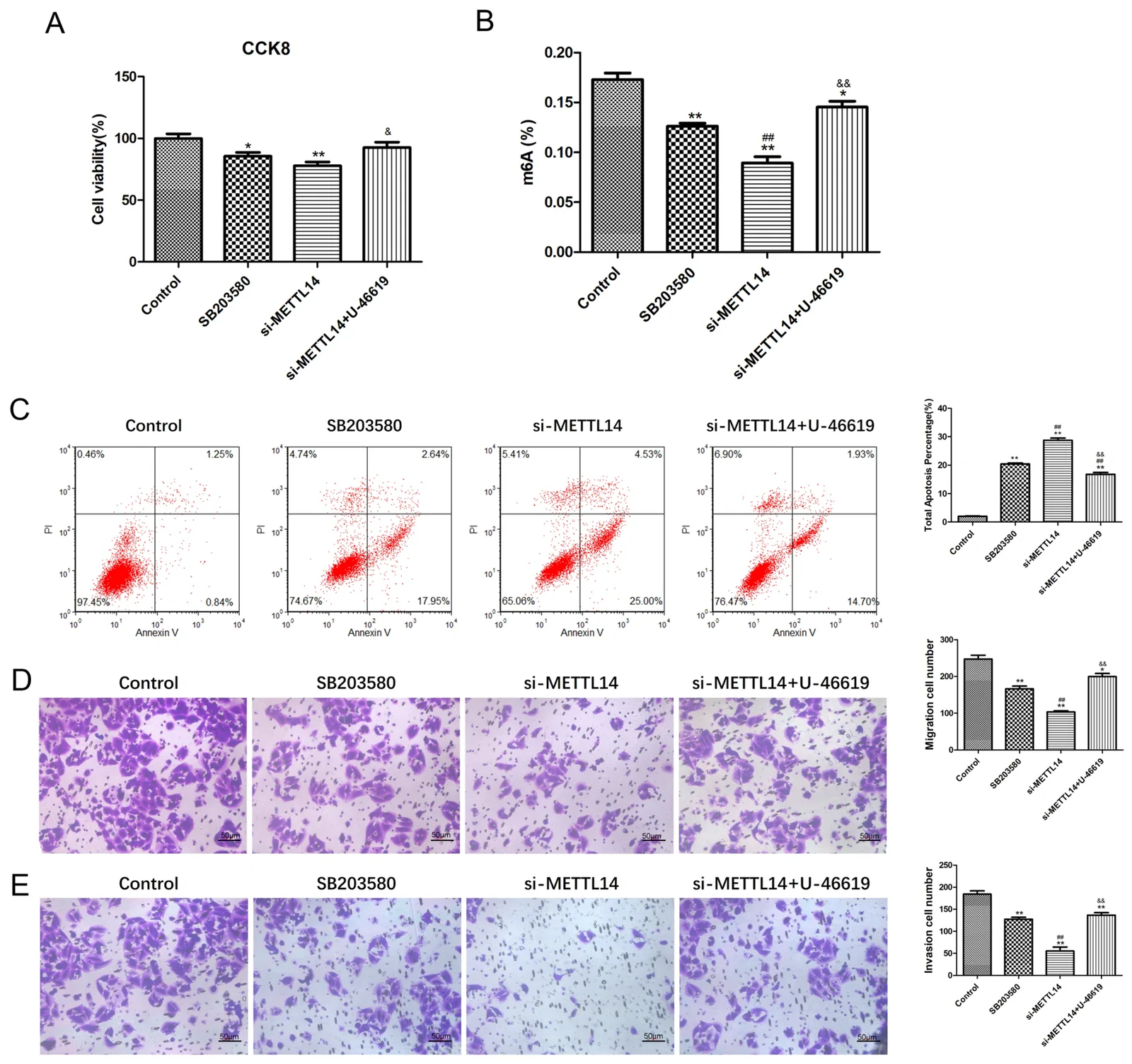
*METTL14* regulates NSCLC cell growth through the MAPK signaling pathway. (**A**) Viability of A549 cells treated with SB203580 (a selective p38 MAPK inhibitor) and U-46619 (a MAPK signaling pathway activator), evaluated using the CCK-8 assay. (**B**) The m^6^A levels in A549 cells treated with SB203580 and U-46619. (**C**) Apoptosis of A549 cells treated with SB203580 and U-46619 evaluated using flow cytometry. The cell migration (**D**) and invasion (**E**) of A549 cells treated with SB203580 and U-46619, assessed using Transwell assay. * *p* < 0.05, ** *p* < 0.01, vs. control; ## *p* < 0.01, vs. SB203580; & *p* < 0.05, && *p* < 0.01, vs. si-*METTL14*.

Eventually, to explore the specific roles of the MAPK signaling pathway in the regulation of A549 cell growth by *METTL14*, we treated A549 cells with SB203580 (a selective p38 MAPK inhibitor) and U-46619 (a MAPK signaling pathway activator). Compared with the control A549 cells, SB203580 treatment, similar to the *METTL14* knockdown, significantly inhibited the viability of cells (*p* < 0.05), whereas U-46619 restored the change in viability caused by *METTL14* knockdown (*p* < 0.05, Figure 6A). The m^6^A levels in cells subjected to different treatments, SB203580 or *METTL14* silencing, were decreased compared with those in the control A549 cells (*p* < 0.01), whereas U-46619 treatment increased the m^6^A levels in the *METTL14* knockdown A549 cells (*p* < 0.01, Figure 6B). These data indicated MAPK–m^6^A positive feedback via upregulation of m^6^A methyltransferases and readers. Although *METTL14*/*MSTRG.292666.16* triggers MAPK signaling, reciprocal crosstalk between MAPK and the m^6^A machinery remains plausible.

Furthermore, similar to that in A549 cells with *METTL14* knockdown, SB203580 significantly enhanced the apoptosis of A549 cells (*p* < 0.01, Figure 6C), while evidently reducing the cell migration and invasion (*p* < 0.01, Figure 6D,E) relative to that in the control A549 cells. However, in comparison with A549 cells with *METTL14* knockdown, U-46619 administration restrained the cell apoptosis (*p* < 0.01, Figure 6C), whereas it increased the cell migration and invasion induced by *METTL14* knockdown (*p* < 0.01, Figure 6D,E).

## 3. Discussion

Tissue and/or blood biomarkers help guide treatment decisions in advanced NSCLC [25]. Abnormal m^6^A RNA methylation is involved in the occurrence, metastasis and prognosis of tumors [26]; however, its role in NSCLC remains unknown. In our prior preliminary work, we established a simple positive correlation between *METTL14* and lncRNA *MSTRG.292666.16* at the phenotypic level, without dissecting the complete downstream signaling cascade or molecular crosstalk [24]. In the present study, we endeavored to resolve this critical gap by delineating a fully integrated oncogenic axis. *METTL14* was significantly upregulated in NSCLC cell lines and exhibited a stable association with lncRNA *MSTRG.292666.16*, enhancing its stability and function. This interaction facilitated the proliferation, migration, and invasion of NSCLC cells while suppressing apoptosis, both in vitro and in vivo. Furthermore, we found that the oncogenic effects of *METTL14*-mediated *MSTRG.292666.16* upregulation were associated with MAPK pathway activation, which is a novel mechanistic insight into NSCLC pathogenesis.

The aberrant expression of *METTL14* in NSCLC cells aligns with previous studies highlighting the dysregulation of m^6^A modifiers in cancer [27]. As a core component of the m^6^A methyltransferase complex, METTL14 has been implicated in various cancers, including colorectal and hepatocellular carcinoma [28,29]. Li et al. [30] demonstrated that as an m^6^A RNA methylase, METTL14 could promote osteosarcoma (OS) progression and resistance to all-*trans* retinoic acid by modulating the stability and translation efficiency of *MN1*, thereby serving as a potential biomarker for prognostic prediction in patients with OS. In another study, *METTL14* silencing suppressed the proliferation, invasion, and migration of lung adenocarcinoma (LUAD) cells in vitro and inhibited tumor growth in vivo [31]. Our results extend these observations to NSCLC, showing that *METTL14* knockdown significantly inhibits tumor growth and metastasis in vitro and in vivo. Additionally, the specific association between *METTL14* and lncRNA *MSTRG.292666.16*, as confirmed via RNA pull-down, RIP, and MeRIP-qPCR assays, underscores the importance of m^6^A modification in regulating lncRNA function. This finding is consistent with emerging evidence that m^6^A modifications can modulate lncRNA interactions with proteins and other RNAs, thereby influencing cancer progression [32].

The oncogenic role of lncRNA *MSTRG.292666.16* in NSCLC was further supported by our functional analyses. In vitro experiments showed that overexpression of lncRNA *MSTRG.292666.16* reversed the inhibitory effects of *METTL14* knockdown on cell viability, migration, and invasion of NSCLC cell lines (A549 and NCI-H1299), while also attenuating their apoptosis. In a previous study, *METTL14* knockdown inhibited the expression of lncRNA *FAS-AS1* in an m^6^A-dependent manner, and *FAS-AS1* overexpression reversed the inhibitory effects of *METTL14* knockdown on JAK/STAT3 signaling and cartilage damage in an osteoarthritis (OA) model, both in vitro and in vivo [33]. Furthermore, lncRNA *OIP5-AS1* overexpression promoted the growth of papillary thyroid cancer (PTC) cells, and *OIP5-AS1* was identified as the downstream target of *METTL14* through RIP and RNA pull-down, which hinted at *METTL14*-mediated *OIP5-AS1* facilitating PTC development [34]. Based on these findings, lncRNA *MSTRG.292666.16* could be inferred to act as a downstream effector of *METTL14*, mediating its protumorigenic effects. Notably, MeRIP-qPCR showed that m^6^A modification of *MSTRG.292666.16* may be *METTL14*-dependent, which can bridge the gap between m^6^A modification and lncRNA function, suggesting that the *METTL14*/lncRNA *MSTRG.292666.16* regulatory axis drives NSCLC.

Our in vitro findings were reinforced by in vivo experiments, demonstrating that *METTL14* knockdown significantly suppressed tumor growth in nude mice, while lncRNA *MSTRG.292666.16* overexpression partially rescued this effect. Histopathological and immunohistochemical analyses revealed reduced proliferation and increased apoptosis in tumors with *METTL14* knockdown, further validating its tumor-suppressive role. The consistency between in vitro and in vivo results highlights the translational potential of targeting *METTL14* and its downstream effectors in NSCLC treatment. Consistent loss- and gain-of-function data from two NSCLC cell lines and subcutaneous xenografts establish the core oncogenic cascade: *METTL14* may deposit m^6^A marks to stabilize *MSTRG.292666.16*, which elevates *MAPK8IP3* and activates ERK1/2, p38, and JNK signaling to fuel NSCLC progression.

The findings that the downregulation of *MAPK8IP3*, p-ERK1/2, p-p38 MAPK and p-JNK after *METTL14* knockdown, and the restoration of their levels upon *MSTRG.292666.16* overexpression is consistent with the well-established role of the MAPK pathway in promoting cell proliferation and survival in NSCLC [35]. *MAPK8IP3*, encoding C-Jun-N-terminal kinase interacting protein 3, is involved in the MAPK signaling pathway and retrograde axonal transport [12,36], and it has been recognized as a novel biomarker in lung cancer research [37]. ERK1/2 are the ultimate components of the MAPK phosphorylation cascade and are indispensable modules among various signaling pathways that shape cellular behavior and fate [38]. Teixeira et al. [39] reported that *FBXO25* could function as a negative regulator of MAPK signal transduction by reducing ERK1/2 activation. As core stress-responsive MAPK branches, p38 MAPK and JNK coordinate cell survival and malignant transformation [40,41]. Their simultaneous suppression upon *METTL14* loss further indicated that the *METTL14-MSTRG.292666.16* axis might activate the entire MAPK cascade rather than selectively targeting ERK alone. Therefore, we speculate that the MAPK pathway may be a key downstream mediator of the *METTL14*/lncRNA *MSTRG.292666.16* regulatory axis. Furthermore, the use of a selective p38 MAPK inhibitor (SB203580) and an activator (U-46619) further confirmed the involvement of this pathway, as their effects mirrored those of METTL14 knockdown and lncRNA MSTRG.292666.16 overexpression, respectively. Notably, U-46619 treatment revealed an unrecognized positive feedback loop: hyperactivated MAPK could reciprocally restore global m^6^A abundance by upregulating key m^6^A writers and readers, forming a self-amplifying oncogenic circuit. However, this conclusion needs to be validated using systematic dual epistatic rescue assays. In a previous study, SB203580 could prevent inflammatory responses and lung injury in a mouse model of acute lung injury induced by lipopolysaccharide [42]. In another study, lncRNA *UCA1* could regulate the mitochondrial dynamics of pancreatic cancer cells through the MAPK/ERK pathway to promote migration, thereby facilitating the progression and prognosis of pancreatic cancer [43]. Combined with our results, we propose that *METTL14*-dependent m^6^A modification of *MSTRG.292666.16* may function as an upstream trigger for MAPK cascade activation to facilitate the growth of NSCLC cells. Our pharmacologic intervention data reveal potential positive feedback signaling from activated MAPK to cellular m^6^A modification machinery, forming a bidirectional regulatory network rather than a simple unidirectional linear pathway.

Our study also sheds light on the broader implications of m^6^A modification in cancer biology. Our in vitro and in vivo experiments showed that *MTLL14* knockdown significantly downregulated IGF2BP1, YTHDC1, and YTHDF1, whereas it upregulated FTO; *MSTRG.292666.16* overexpression could revert the *METTL14* knockdown–induced effect on the expression. IGF2BP1, YTHDC1, and YTHDF1 are m^6^A readers, whereas FTO is one of the m^6^A erasers [27]. YTHDF2, which is crucial for m^6^A modification, could mediate the degradation of *TINCR* and *METTL14* could restrain pyroptosis and diabetic cardiomyopathy by increasing the m^6^A modification levels of lncRNA *TINCR* and thereby downregulating it [44]. Furthermore, FTO is upregulated in HCC, and targeting FTO can stabilize the degradation of *GPNMB* by reducing the m^6^A abundance of *GPNMB*, thereby inhibiting tumorigenesis in HCC and enhancing the immune response [45]. Three non-exclusive mechanisms may explain this correlation: MAPK feedback validated by U-46619 treatment, nonspecific shifts secondary to impaired cell growth/apoptosis, and unconfirmed direct *METTL14*-dependent regulation of these modifiers. In the absence of epistasis and binding assays that can help assess these possibilities, we refrain from defining a specific *METTL14*–m^6^A regulatory axis. Further experiments are needed to resolve the hierarchical crosstalk among these putative mechanisms. Taken together, the dynamic regulation of m^6^A levels by *METTL14* and its impact on lncRNA *MSTRG.292666.16* function underscore the complexity of epitranscriptomic mechanisms in NSCLC. The interplay between m^6^A modifiers, lncRNAs, and signaling pathways such as MAPK opens new avenues for research into cancer therapeutics. For instance, small molecules targeting *METTL14* or lncRNA *MSTRG.292666.16* can be developed to disrupt this oncogenic axis.

This study had some limitations. First, all mechanistic and functional experiments were conducted in A549 and NCI-H1299 cells and in xenograft mouse models, without validation in human NSCLC clinical tissues with full clinical and survival data, as we have not yet built a paired patient cohort to analyze correlations between *METTL14*, *MSTRG.292666.16*, *MAPK8IP3*, and p-ERK1/2 or performed survival analysis. Second, *MSTRG.292666.16* originated from our in-house RNA-seq data; BLAST (https://blast.ncbi.nlm.nih.gov; Access date: 22 July 2025) searches revealed no matching annotated transcripts in The Cancer Genome Atlas (TCGA) lung datasets, precluding TCGA-based expression, correlation, and survival analyses. In follow-up prospective studies, we intend to collect matched tumor and paratumor NSCLC samples and perform RNA quantification, IHC, and long-term survival tracking to systematically verify the clinical prognostic significance of the *METTL14/MSTRG.292666.16/MAPK* axis. Third, we only utilized subcutaneous A549 xenografts (*n* = 6 per group) for in vivo assays. This ectopic model cannot recapitulate the native lung microenvironment or NSCLC invasive/metastatic traits. As such, our in vivo findings are limited to primary tumor growth. Orthotopic and metastatic models will be used in future studies to fully dissect lung cancer progression. Additionally, U-46619 treatment revealed MAPK-mediated feedback regulation of global m^6^A abundance. However, systematic epistatic dual-intervention rescue assays were not conducted to define the upstream–downstream hierarchy between m^6^A modifiers and MAPK signaling. Dual-manipulation functional tests will be performed in follow-up research to dissect this regulatory cascade.

In conclusion, our findings establish *METTL14* as a critical regulator of lncRNA *MSTRG.292666.16* through m^6^A modification, driving NSCLC growth via the MAPK pathway. These results deepen our understanding of the molecular mechanisms underlying NSCLC and lay the foundation for targeting the *METTL14/MSTRG.292666.16/MAPK* axis as a promising therapeutic strategy for NSCLC. Future studies should explore the clinical applicability of targeting this axis, particularly in patients with advanced or treatment-resistant NSCLC. Additionally, investigating the interplay between *METTL14* and other lncRNAs or signaling pathways can further unravel the complexities of NSCLC pathogenesis and uncover novel therapeutic opportunities.

## 4. Materials and Methods

### 4.1. Cell Culture and Transfection

Human bronchial epithelial cell line, BEAS-2B, and human NSCLC cell lines, A549, NCI-H1299, and NCI-H1650, were obtained from Cell bank, Chinese Academy of Sciences (Shanghai, China). All the cells were cultured in RPMI 1640 medium (Thermo Fisher Scientific, Waltham, MA, USA) supplemented with 10% fetal bovine serum (FBS, Thermo Fisher Scientific) and 1% penicillin/streptomycin (Thermo Fisher Scientific) in an incubator with 5% CO_2_ at 37 °C.

A549 and NCI-H1299 cells with *METTL14* knockdown and *MSTRG.292666.16* overexpression were established using si-*METTL14* and OE-lncMSTRG, as previously reported [25]. Briefly, the si-negative control (NC), si-METTL14, OE-NC and OE-lncMSTRG were designed and obtained from Generay (Shanghai, China). A549 and NCI-H1299 cells were seeded in 24-well plates at the density of 4 × 10^4^ cells/well, and cultured overnight. Thereafter, the medium was changed to serum-free medium, and the cells were transfected with 15 pmol si-NC or si-*METTL14* and 1 μg OE-NC or OE-lncMSTRG using Lipofectamine 3000 (Thermo Fisher Scientific) in accordance with manufacturer’s instructions. After 6 h of transfection, the medium was replaced with complete medium and the culture was continued for another 48 h. Thereafter, total RNA was isolated from the cells to measure the expression of *METTL14* and *MSTRG.292666.16* using RT-qPCR to evaluate the cell transfection efficiency. The sequences of *METTL14* and *MSTRG.292666.16* are presented in Table 1.

### 4.2. Cell Viability and Apoptosis Assays

The viability profiles across A549 and NCI-H1299 cell transfection variants were evaluated using the CCK-8 kit (Beyotime Biotechnology, Shanghai, China). Briefly, A549 and NCI-H1299 cells were seeded in the wells of 96-well plates at a density of 1 × 10^4^ cells/well and cultured for 24, 48 and 72 h. Thereafter, 10 μL of CCK-8 solution was added to each well. After 2 h of incubation, the absorbance at 450 nm was detected using a microplate reader.

Cell apoptosis was assessed using an Annexin V-FITC/PI detection kit (Beyotime Biotechnology), following manufacturer’s instructions. Briefly, A549 and NCI-H1299 cells subjected to various treatments were collected, centrifuged (1000 rpm for 5 min), and resuspended in 195 μL binding buffer. After adding 5 μL Annexin V-FITC and 5 μL PI, samples were incubated at room temperature in the dark for 20 min. Cell apoptosis was determined using a flow cytometer.

### 4.3. Cell Migration and Invasion Assays

Cell migration and invasion were assessed using 8 µm pore-size Transwell chambers (Guangzhou Jet Bio-Filtration Co., Ltd., Guangzhou, China). For cell invasion assays, Transwell chambers were precoated with a layer of Matrigel (extracellular matrix gel, Corning Inc., Corning, NY, USA). Briefly, cells subjected to different transfections were harvested and inoculated into the upper inserts of the chambers, while the lower chambers were filled with complete medium. Following 48 h of incubation, the cells were washed twice with PBS and fixed with 4% paraformaldehyde at room temperature for 20 min. After washing, the cells were stained with 0.1% crystal violet by incubating them with the dye at room temperature for 20 min. Excess dye was removed, and the chambers were air-dried before cell images were captured under an inverted microscope, and relative cell numbers were analyzed.

### 4.4. Animal Experiments

A549 cells (transfected with si-NC, si-*METTL14*, and si-*METTL14*+OE-lncMSTRG) in the logarithmic growth phase were washed and digested with 0.25% trypsin for 2 min. The digestion was terminated by adding FBS to prevent over-digestion. After low-speed centrifugation (1000 rpm, 5 min), the cells were collected, washed twice with PBS, and resuspended in serum-free medium. Following homogenization, the concentration of the cells was adjusted to 5 × 10^7^ cells/mL using sterile PBS.

Eighteen 4–6-week-old male BALB/c male nude mice (SPF grade) were procured from SLAC Laboratory Animal Co., Ltd. (Shanghai, China) and housed under controlled conditions (24 ± 2 °C, 50 ± 5% relative humidity, 12 h light/dark cycle) with unrestricted access to food and water. After a 7-day acclimation period, the mice were randomly assigned to three groups (*n* = 6 per group)—si-NC, si-*METTL14*, and si-*METTL14*+OE-lncMSTRG—which were subcutaneously injected with 100 μL (5 × 10^7^ cells/mL) of si-NC-transfected, si-*METTL14*-transfected, and si-*METTL14*+OE-lncMSTRG-transfected A549 cells, respectively, into the right forelimb axilla. After tumor implantation, the mental state, activity, diet, urine, and fecal excretion of the mice were monitored daily. The longest and shortest diameters of subcutaneous tumors were measured weekly using vernier calipers. Subcutaneous tumor volume (V) was calculated using the formula: V (mm^3^) = (longest tumor diameter (mm) × shortest diameter (mm) × shortest diameter (mm)) × 0.5. Based on the obtained tumor volumes, a growth curve was plotted for the transplanted tumors. After 5 weeks of feeding, the mice were euthanized via cervical dislocation, and tumors were excised, cleaned, and photographed. Additionally, a portion of the tumor tissues was fixed with 4% paraformaldehyde for further histopathological and immunohistochemistry (IHC) analyses, and the other tissues were used for investigating the molecular mechanism. All the animal procedures complied with the guidelines of the National Medical Advisory Committee (NMAC) and were approved by the Institutional Animal Care and Use Committee of Shanghai Pulmonary Hospital, Tongji University School of Medicine (approval no. K25-471Y).

### 4.5. Histopathological and IHC Analyses

The fixed lung tissues were embedded in paraffin and sectioned into 4 µm-thick sections. The sections were deparaffined in xylene and rehydrated by passing through a descending ethanol gradient. The sections were stained with hematoxylin for 10 min and then with eosin for 90 s. After dehydration through an alcohol gradient, clearing in xylene, and mounting with neutral resin, the stained sections were observed under a light microscope (ECLIPSE E100, Nikon, Tokyo, Japan).

For IHC analysis, the sections were deparaffinized in xylene, rehydrated by passing through a descending ethanol series, and underwent heat-induced antigen retrieval using ethylene diamine tetraacetic acid (EDTA) buffer. The sections were blocked with 5% bovine serum albumin and then sequentially incubated with anti-Ki67 antibody (1:400, Servicebio, Wuhan, China) overnight at 4 °C and with HRP-labeled goat anti-rabbit IgG for 1 h. This was followed by incubation with DAB chromogenic solution (containing 0.05% DAB and 0.01% H_2_O_2_) for 3 min and nuclear staining with hematoxylin for 30 s. The sections were then sealed with neutral gum and imaged under a fluorescence microscope (IX70, Olympus, Tokyo, Japan). The images were analyzed using ImageJ software (version 1.54f).

### 4.6. TUNEL Assay

A One Step TUNEL Apoptosis Assay Kit (Beyotime Biotechnology) was used to determine the cell apoptosis in tumor tissues in accordance with the manufacturer’s instructions. Briefly, tissue sections were deparaffinized with xylene, rehydrated through a gradient ethanol series, and then permeabilized with 0.3% Triton X-100 for 5 min. After washing, the sections were incubated with a reaction mixture containing terminal deoxynucleotidyl transferase (TdT enzyme) and fluorescently labeled dUTP in a dark humid chamber at 37 °C for 60 min. For negative control, TdT enzyme was replaced with PBS in the reaction mixture. After washing, the sections were overlaid with 1 mL 1× Hoechst 33342 solution, incubated for 10 min in the dark, and mounted with an anti-fluorescence quenching mounting medium. The sections were visualized under a fluorescence microscope and imaged; green fluorescence indicated apoptotic cells and blue fluorescence was indicative of stained nuclei.

### 4.7. RT-qPCR

Total RNA was isolated from cells or tissue samples using an RNA extraction kit (Servicebio). The quality and concentration of the extracted RNA were assessed using a microplate reader (Thermo Fisher Scientific). Subsequently, 1 μg of RNA was reverse-transcribed into cDNA using the PrimeScript™ II 1st Strand cDNA Synthesis Kit (Takara Bio Inc., Kusatsu, Shiga, Japan). RT-qPCR was performed with PerfectStart^®^ Green qPCR SuperMix (TransGen Biotech, Beijing, China) according to the manufacturer’s specifications, using primers, details of which are provided in Table 1. Relative gene expression was calculated using the 2^−ΔΔCt^ method, with expression normalized to that of the endogenous control *GAPDH*.

### 4.8. Western Blotting

Total proteins were extracted from tissue samples using RIPA lysis buffer (Beyotime Biotechnology) and quantified using a bicinchoninic acid (BCA) assay kit (Beyotime Biotechnology). The proteins were separated via sodium dodecylsulfate–polyacrylamide gel electrophoresis and blotted onto polyvinylidene difluoride membrane. The blots were blocked with 5% non-fat milk and incubated overnight at 4 °C with primary antibodies against METTL14 (1:2000, Proteintech, Wuhan, China), FTO (1:1000, Proteintech), IGF2BP1 (1:5000, Proteintech), YTHDC1 (1:800, Proteintech), YTHDF1 (1:800, Proteintech), MAPK8IP3 (1:500, Proteintech), p-ERK1/2 (1:1000, Proteintech), t-ERK1/2 (1:2000, Proteintech), and GAPDH (1:10,000, Proteintech). The membranes were then probed with species-matched HRP-conjugated secondary antibodies (1:10,000, Jackson ImmunoResearch, West Grove, PA, USA) for 2 h at 37 °C. Protein signals were detected using a chemiluminescence imaging system (Beyotime Biotechnology, Shanghai, China) and quantified using ImageJ software.

### 4.9. Detection of Global m^6^A Modification

Total RNA was extracted from cells or tissue samples using the RNAiso Plus kit (Trizol, Takara) following the manufacturer’s instructions. RNA purity and concentration were determined using a microplate reader (Thermo Fisher Scientific). Subsequently, the levels of m^6^A modification in NSCLC cells and tissue samples were determined using an m^6^A RNA Methylation Quantification Kit (Epigentek Group Inc., Farmingdale, NY, USA), according to the kit manual.

### 4.10. RNA Pull-Down and RIP Assays

In the RNA pull-down assay, RNAs were synthesized via in vitro transcription using T7 RNA polymerase and simultaneously labeled with biotin using the T7 Biotin Labeled RNA Synthesis Kit (Geneseed Biotech Co., Ltd., Guangzhou, China) employing linearized pCDNA3.1(+)-lncMSTRG plasmid as a template. Subsequently, RNA–protein pull-down was conducted using the Pierce™ Magnetic RNA-Protein Pull-Down Kit (Thermo Fisher Scientific). The biotinylated RNA was captured with streptavidin magnetic beads. Cell lysates were mixed with the biotinylated RNA by rotation at 4 °C for 1 h. The magnetic beads were washed with SDS buffer and boiled to recover the bound proteins, which were then detected via Western blot analysis with antibodies against METTL3 (1:5000, Proteintech), METTL14 (1:2000, Proteintech), WTAP (1:5000, Proteintech), and β-action (1:10,000, Proteintech).

The RIP assay was performed using A549 and NCI-H1299 cells with an RNA Immunoprecipitation Kit (Geneseed Biotech Co., Ltd.), as per the manufacturer’s instructions. Briefly, the cells were lysed with RIP lysis buffer (Millipore, Burlington, MA, USA), and the cell extracts were incubated overnight with IgG (1:200, Abcam, Cambridge, UK) and METTL14 (1: 2000, Cell Signaling Technology, Danvers, MA, USA) antibodies at 4 °C. The coprecipitated proteins were detected using Western blotting, and the total RNA was used to determine the fold enrichment of *MSTRG.292666.16* via RT-qPCR.

### 4.11. Statistical Analysis

Data are represented as mean ± standard deviation of values from three independent experiments. Statistical analyses were performed using GraphPad Prism 9.0 software (San Diego, CA, USA). For comparing differences between two groups, unpaired *t*-test was applied, whereas differences among multiple groups (≥3) were analyzed using one-way analysis of variance followed by Tukey’s test. A value of *p* < 0.05 was considered to indicate statistical significance.

## Figures and Tables

**Table 1:** The sequences of all primers.

Primer	Sequence (5′-3′)
*MSTRG.292666.16*	F: CTGGAGTGCAGTGGCTATTC
	R: AGGCTGAGGTGGGAGGAT
*MAPK8IP3*	F: GTGTACCAGGACGACTACTGC
	R: GCACCGAGTCTAGGTTCTCCA
*METTL14*	F: AGTGCCGACAGCATTGGTG
	R: GGAGCAGAGGTATCATAGGAAGC
*METTL3*	F: TTGTCTCCAACCTTCCGTAGT
	R: CCAGATCAGAGAGGTGGTGTAG
*WTAP*	F: CTTCCCAAGAAGGTTCGATTGA
	R: TCAGACTCTCTTAGGCCAGTTAC
*GAPDH*	F: TGACAACTTTGGTATCGTGGAAGG
	R: AGGCAGGGATGATGTTCTGGAGAG

## Data Availability

The original contributions presented in this study are included in the article. Further inquiries can be directed to the corresponding authors.
